# How information literacy influences creative skills among medical students? The mediating role of lifelong learning

**DOI:** 10.1080/10872981.2023.2176734

**Published:** 2023-03-19

**Authors:** Muhammad Asif Naveed, Javed Iqbal, Muhammad Zaheer Asghar, Rozeen Shaukat, Rabia Kishwer

**Affiliations:** a Department of Information Management, University of Sargodha, Sargodha, Pakistan; b School of Education, Guangzhou University, Guangzhou, Guangdong, China; c School of Education, Huazhong University of Science and Technology, Wuhan, China; d Faculty of Teacher Education, University of Helsinki, Helsinki, Finland; e Faculty of Educational Sciences, University of Helsinki, Helsinki, Finland; f University of Management and Technology, Lahore, Pakistan; g Office of Research Innovation and Commercialization, University of Management and Technology, Lahore; h International Islamic University, Islamabad, Pakistan; i Faculty of Education, International Islamic University, Islamabad, Pakistan

**Keywords:** Information literacy, lifelong learning, creativity, medical students, Pakistan

## Abstract

This research investigated the influence of information literacy on the creative skills of medical students through the mediation of lifelong learning. A cross-sectional survey was conducted for data collection among medical students, recruited through a stratified convenient sampling procedure, of Khyber Pakhtunkhwa, Pakistan. The questionnaire was personally administered by visiting each college with written permission. A total of 473 survey responses were collected and analysed using the partial least squares structural equation modelling. The results revealed that the information literacy of medical students had a direct and indirect but positive influence on their creative skills. Lifelong learning not had a direct but positive effect on creative skills but also mediated the relationship between information literacy and creative skills. The empirical evidence for how information literacy influences creative skills through the mediated role of lifelong learning may inform policy and practice for information literacy instructions. These results may help academicians and information specialists to initiate a credited or integrated course on information literacy in the curriculum of medical students not only in Pakistan but also in other developing countries. This research would be a worthwhile contribution to the existing research on information literacy as the mediated role of lifelong learning between information literacy and creative skills has never been examined so far.

## Introduction

Educational institutions aim to prepare their students as independent, self-regulated, confident, and lifelong learners equipped with creative and entrepreneurial capabilities [1–4]. In this regard, information literacy has been considered as a catalyst for educational change [5]. Information literacy refers to a set of abilities enabling people in recognizing their information needs, where to find it, and how to evaluate it, use and create ethically to achieve their personal and professional goals [6]. CILIP [7] defined information literacy as ‘the ability to think critically and make balanced judgments about any information we find and use. It empowers us as citizens to develop informed views and to engage fully with society.’ Information literacy plays an important role in the capacity building of students to undertake information related tasks, make the best use of information and adopt appropriate information behaviour to achieve educational and career goals [8]. It also influences positively students’ academic engagement and academic performance [9–11].

In the information age, students are required to develop creative skills to survive and compete for employability in modern enterprises to achieve organizational goals, viable competitive advantage, and improve organizational performance and effectiveness [12–14]. Such skills enable individuals for making balanced decisions and application of information to a creative task at hand [15]. Creativity refers to the production of unique ideas or products by using intelligence, prior knowledge, and imagination [16]. Creativity does not occur randomly but as a result of a process that is composed of periods such as preparation, incubation, enlightenment, and verification [17]. It also included processes such as problem identification, sense-making of elements, gathering needed information, and learning varied approaches to the problem [18]. The process of creativity requires certain skills for recognizing the need for information related to a problem at hand, and how to find, access, evaluate, use, and manage it in an ethical and legal manner [19]. Such skills are labelled as information literacy enabling individuals in recognizing information needs and adopting the appropriate information seeking behaviour for a given task [20]. Therefore, it is quite legitimate to assume that information literacy lays the foundation for creativity and may influence creative skills. Few existing studies from the academic context also reported a strong and positive relationship between students’ information literacy and creativity [21–23]. Conclusively, the empirical evidence is still inadequate, the relationship between information literacy with creative skills justifies further investigation.

With the rapid and continuous technological change, it is necessary to keep one’s self updated on recent developments to find creative solutions for problems at hand and produce creative products to enhance employability 24] and obtain a sustainable competitive advantage [16]. In this regard, the development of lifelong learning habits among students is necessary to keep up with technological advancements and transformation. Lifelong learning refers to an ongoing, voluntary, and self-initiated pursuit of knowledge and skills acquisition [25,26]. According to Plaza and Robotham [27], lifelong learning is essential for the creation and maintenance of positive attitudes toward knowledge acquisition and application either for personal or professional development. On the other hand, Horton [28] argued that information literacy and lifelong learning are largely self-motivated, self-directed, and self-empowered, which need to be ideally harnessed together to improve the quality of learning and set up multiple choices in different contexts. In addition, both the concepts had a strategic, mutual, and reinforcing relationship with one another that is essential for success at an individual, organizational and societal level [29,30]. The positive association of information literacy with lifelong learning has also been reported by some empirical studies [25,31–33]. Furthermore, An inquiry by Kesici [16] also reported that lifelong learning disposition is the positive predictor of creative thinking skills among Turkish teachers. Keeping this in mind, it was quite logical to empirically investigate the relationship of between information literacy and lifelong learning.

Creativity also requires a lifelong effort as the production of new, original, and valuable ideas is not achieved in one go. Tsai [34] argued that creativity is not actualized through a single-time effort as it is a result of a long process of learning. It is an important skill today which is required to be developed through education [35,36]. All types of learning, both formal and informal, are usually considered within the scope of lifelong learning. The primary purpose of lifelong learning is compliance with new developments, acquisition of new knowledge, and usage of that knowledge to prevent people from life alienation [37]. The adjustment of students with contemporary developments indicated that the future workforce is prepared to solve real life issues. The efforts that are put together for creativity can be described as lifelong learning. An inquiry by Kesici [16] also reported that lifelong learning disposition is the positive predictor of creative thinking skills among Turkish teachers. Hence, it can be inferred that lifelong learning is also one of the factors contributing to the development of creative skills.

Considering the lack of existing empirical evidence, the interrelationships between information literacy, creative skills, and lifelong learning merits further investigation. No study appeared to have been conducted on the interrelationship between information literacy, creative skills, and lifelong learning, particularly among medical students. Therefore, this study aimed to examine the interrelationship of information literacy, creative skills, and lifelong learning among medical students of Khyber Pakhtunkhwa, Pakistan. Understanding the interrelationships between information literacy, creative skills, and lifelong learning among medical students would help educational institutions and healthcare organizations in recognizing the way information literacy influences in academia and the workplace. This study addressed specifically the following research questions:

### Research questions (RQs)

1. How does information literacy influence creative skills among medical students?
2. How does information literacy of medical students influence their lifelong learning?
3. How does lifelong learning of medical students influence their creative skills?
4. How does lifelong learning mediate the relationship between information literacy and creative skills?

### Research hypotheses

H_1_: Information literacy positively influence creative skills.

H_2_: Information literacy positively influences lifelong learning.

H_3_: Lifelong learning positively influences creative skills.

H_1_ ’: Lifelong learning positively mediates the relationship between information literacy and creative skills.

## Methodology

### Research model

**Figure 1.**
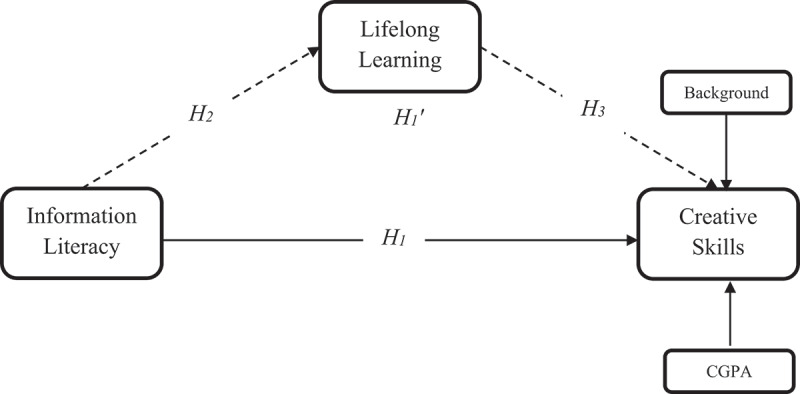
Proposed research model.

The purpose of this study was to investigate the interrelationships of information literacy, lifelong learning, and creative skills of medical students. as outlined in the proposed research model delineating the hypothesized relationships between these constructs (Figure 1).

### Design and method

A quantitative research design using a survey method was deployed to investigate the interrelationships of information literacy, lifelong learning, and creative skills among medical students in the province of Khyber Pakhtunkhwa, Pakistan. A structured questionnaire was utilized to collect data from medical students as it was useful to collect responses from a larger and geographically dispersed population. The questionnaire comprised 36 statements related to information literacy (23 statements), lifelong learning (5 statements), and creative skills (8 statements) along with certain demographic variables such as age, gender (male and female), geographical background (rural and urban) and CGPA.

### Measures

#### Information literacy

The 23 items related to information literacy were adopted from the Information Literacy Self-Efficacy Scale developed by Kurbanoglu et al. [38]. The instances of such statements included ‘I can identify a variety of potential sources of information’, ‘I can use many resources at the same time to make research’, and ‘I can evaluate information critically’ (See Appendix 1). Each statement was measured on a seven-point Likert scale ranging from ‘always almost true’ to ‘almost never true’ (e.g., 7 = Almost Always True, 6 = Usually True, 5 = Often True, 4 = Occasionally True, 3 = Sometime but Infrequently True, 2 = Usually Not True, 1 = Almost Never True). The reliability analysis in Table 2 indicated higher reliability for the measure of information literacy as there were high values for the Cronbach alpha (CA = 0.937) and composite reliability (CR = 0.943). The CA value ranges between zero and one. The value of CA near to one exhibits higher reliability and near to zero indicates lower reliability [39]. While the value of CR should be slightly higher than CA [40]. In addition, the reliability of this scale (e.g., CR = 0.938 CA = 0.929) was also reported by Soroya et al. [41] with medical students. Richardson [42] also reported its suitability with Belgian medical students as the values of CA were greater than 0.70 for overall scale and each of its sub-scales. The certain other studies also reported the reliability and validity of this scale with other students from varied disciplines, geographical and cultural contexts [43,44]

#### Lifelong learning

The 5-statements measuring lifelong learning were adopted from the work of Kirby et al. [45]. The instances of such statements included ‘I feel I am a self-directed learner.’, ‘I try to relate academic learning to practical issues’, and ‘It is my responsibility to make sense of what I learn at school’. This scale was particularly designed for students rather than professionals or teachers. Furthermore, it was of reasonable length which can easily be used with other variables (See Appendix 1). Each statement was measured on a five-point Likert scale ranging from ‘strongly agree’ to ‘strongly disagree’ (e.g., 5 = strongly agree, 4 = agree, 3 = undecided, 2 = disagree, 1 = strongly disagree). Table 2 also indicated the higher reliability for the measure of lifelong learning as the values of CA = 0.737 and CR = 0.826 were reasonable and acceptable. This scale was also reported as reliable and valid with chemical engineering students in Malaysia by Yap and Tan [46] as the value of CA (0.67) was in the acceptable range.

#### Creative skills

The creative skills of these medical students were measured using eight statements adopted from the creativity scale developed by Muñoz-Doyague et al. [47]. Examples of such statements included ‘I usually search out new technologies, processes, techniques, and ideas’, ‘I use existing information to develop ideas that are useful for my academic performance’, and ‘I develop ideas, methods or processes that are both original and useful for my career’ (See Appendix 1). These statements were also measured on a five-point Likert scale ranging from ‘strongly agree’ to ‘strongly disagree’ (e.g., 5 = strongly agree, 4 = agree, 3 = undecided, 2 = disagree, 1 = strongly disagree). The figures in Table 2 also exhibited higher reliability for this measure as there were acceptable values for CA = 0.826 and CR = 0.868.

### Population and sampling

All the medical students having enrolment in third and fourth year of MBBS at medical colleges of Khyber Pakhtunkhwa were considered as the population for this study. The minimum sample size calculated for an unknown size of the population was 384 based on the 95% confidence level and five percent margin of error. The medical students were recruited from 22 medical colleges in the province of Khyber Pakhtunkhwa, Pakistan. The recruitment of the survey participants for each medical college was made through a stratified convenient sampling process as it was not possible through a random process due to the non-availability of lists, accessibility issues, and time limitations.

### Data collection and analysis

The researchers personally visited each college with written permission from the concerned authorities for data collection. The questionnaires were administered by visiting the classrooms among medical students of each college. The medical students were asked to fill voluntarily the printed questionnaire and assured of confidentiality and anonymity of the provided data. A total of 473 survey responses were collected in approximately two-three months of data collection and entered carefully into computer software for data analysis. The PLS-SEM (partial least squares structural equation modelling) was deployed for the measurement of direct and indirect relationships between information literacy, lifelong learning, and creative skills as proposed in the research model through SmartPLS 3.3.3 version. Initially, the reliability and validity of the used scales were established through measurement modelling such as factor loading, Cronbach Alpha, roh_A, and convergent validity and discriminant validity. Afterward, the structural mediation modelling was applied to measure the direct and indirect relationship between these constructs.

## Results

### Demographic characteristics

Table 1 outlined the demographic characteristics of the survey participants. Of the 473 survey respondents, a majority (*n* = 291, 61.50%) of these medical students had an age bracket of 21–23 years, followed by those students having up to 20 years (*n* = 89, 18.80%) and 24–26 years (*n* = 82, 17.40%). Only 11 students (2.1%) had their age larger than 26 years. There were 246 (52.00%) males and 227 (48.00%) females. As far as their geographical background is concerned, 248 (51.60%) students belonged to urban areas and 229 (48.40%) came from rural areas. Concerning the distribution of their CGPA (Cumulative Grading Point Average), a large majority (*n* = 390, 82.5%) of these medical students had a CGPA bracket of 3.1–3.5 which was followed by those having CGPA less than 3.0 (*n* = 43, 9.0%) and greater than 3.50 (*n* = 40, .50%).

**Table 1.** The demographic composition of the medical students (*N* = 473).

Characteristics	Categories	Frequency (n)	Percentage (%)
Age (Years)	Up to 20	89	18.80
	21–23	291	61.50
	24–26	82	17.40
	26+	11	2.21
Gender	Male	246	52.0
	Female	227	48.0
Geographical Background	Urban	244	51.6
	Rural	229	48.4
CGPA	Up to 2.5	1	.2
	2.5–3.0	42	8.8
	3.1–3.50	390	82.5
	3.51–4.0	40	8.50

### The measurement model

The measurement model analysis was applied to assess the validity and reliability of each instrument through SmartPLS 3.3.9. In addition, the measurement of structural relations among latent variables was completed through structural equation modeling (SEM) using SmartPLS as it is more statistically effective, efficient, and less sensitive software towards sample size as compared to other software used for covariance-based SEM, such as AMOS [48]. This research examined the influence of information literacy on creativity through the mediation of lifelong learning among medical students in Khyber Pakhtunkhwa, Pakistan. Before the measurement of interrelationships between these variables, the reliability and validity of the measures used in the present research were ensured (Table 2).

**Table 2.** Reliability and validity analysis.

Scales	Factor Loading	Cronbach’s Alpha	rho_A	Composite Reliability	Average Variance Extracted (AVE)
Information Literacy		0.937	0.938	0.943	0.521
IL1	0.657				
IL2	0.618				
IL3	0.605				
IL4	0.623				
IL5	0.632				
IL6	0.717				
IL7	0.676				
IL8	0.675				
IL9	0.647				
IL10	0.653				
IL11	0.693				
IL12	0.667				
IL13	0.649				
IL14	0.648				
IL15	0.618				
IL16	0.632				
IL17	0.621				
IL18	0.631				
IL19	0.653				
IL20	0.698				
IL21	0.619				
IL22	0.617				
IL23	0.659				
Lifelong Learning		0.737	0.738	0.826	0.588
LL1	0.717				
LL2	0.687				
LL3	0.734				
LL4	0.65				
LL5	0.703				
Creative Skills		0.826	0.827	0.868	0.551
CS1	0.668				
CS2	0.68				
CS3	0.619				
CS4	0.649				
CS5	0.701				
CS6	0.684				
CS7	0.662				
CS8	0.706				

**Figure 2.**
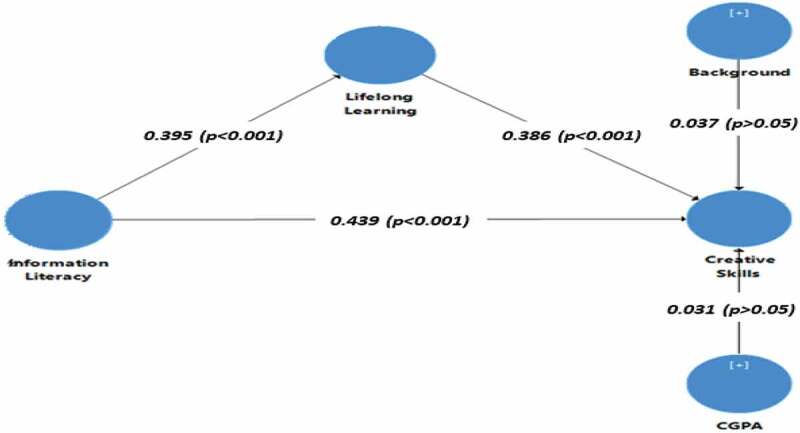
Research model.

Table 2 showed the reliability and validity of all the measures used in the present study. The key reliability indicators included: factor loading, Cronbach’s alpha, rho_A, and composite reliability. According to Hair et al. [48], the rule of thumb for the strength of factor loading is the standard value larger than 0.60. These figures indicated the appropriate strength for the factor loading of all the statements as all the values were greater than 0.60. Furthermore, the thresh hold value for the Cronbach’s alpha, rho_A, and composite reliability indicators was 0.70 (authors). These figures also indicated that values for the Cronbach’s alpha, rho_A, and composite reliability indicators were greater than the threshold values which meant the measures used in the present research met the reliability requirements. The convergent validity of these measures was ensured by applying average variance extracted (AVE). The standard value for AVE is 0.50 [49,50]. Table 2 showed a higher value of AVE for all these constructs than the threshold value. Thus, it can be concluded that the measures used in the present research met the criterion of reliability and validity to collect the data (See Table 2 and Figure 2).

The discriminant validity in partial least squares-structural equation modeling (PLS-SEM) was measured using the heterotrait-monotrait (HTMT) approach as recommended by Henseler et al. [51]. The HTMT approach is considered more appropriate to measure the discriminant validity in PLS-SEM than the approach suggested by Fornell and Larcker [52]. They further explained that Fornell and Larcker [52] approach is an unreliable method to measure the discriminant validity [51]. The HTMT approach is referred to as the ‘item correlation among constructs with the correlations within items of the same construct’. Henseler et al. [51] proposed a less than 0.90 HTMT threshold value which explains that the constructs were proved discriminately valid. Table 3 showed that the measures such as information literacy, lifelong learning, and creativity met the requirements of discriminant validity.

**Table 3.** Discriminant validity.

Constructs	Creative Skills	Information Literacy	Lifelong Learning
Creative Skills	0.672		
Information Literacy	0.597	0.649	
Lifelong Learning	0.566	0.395	0.699

The collinearity issue must be resolved during the SEM analysis. The Variance Inflation Factor (VIF) technique was applied to resolve the collinearity issue. The value of VIF less than 5 and 3 is considered ideal and threshold [53]. Table 4 showed the collinearity analysis and model fit. These figures indicated the range of VIF values among dimensions is 1.000 to 1.201, indicating no collinearity problems among information literacy, lifelong learning, and creativity dimensions.

**Table 4.** Collinearity and model fit.

Dimensions	CS-VIF	LL-VIF	Model Fit	
Creative Skills (CS)			RSMS	0.063
Information Literacy (IL)	1.189	1.000	NFI	0.871
Lifelong Learning (LL)	1.201		RMS_Theta	0.107

The three main model fit indicators, namely, SRMR, NFI, and RMS_ theta are usually employed in PLS-SEM analysis to measure the overall fitness of the model. The threshold value for SRMR is less than 0.08. The SRMR value in Table 4 indicates is 0.063 which means that the model was well fit. Similarly, The NFI ideal threshold value is 1. The near to 1 value is considered a better model fit. Table 4 also indicates an acceptable value for NFI, which is 0.871, indicating a better model fit. The RMS_theta indices are used for reflective measurement modeling. The threshold value for RMS_theta indices is less than .12. Table 4 also indicates that the value RMS_theta value is 0.107 which means that model is fit and appropriate. Concussively, the model proposed in the present research was shown to be pretty well suited in general.

The model explanatory power is measured through the R^2^ value which ranges from 0 to 1. The greater value of R^2^ value indicates greater explanatory power. The threshold values for R^2^ of the model up to 0.25, 0.50, and 0.75 are considered weak, moderate, and strong respect for the explanatory power. Table 5 shows that latent variables such as creative skills (R^2^ value, 0.487) and lifelong learning (R^2^ value, 0.356) have explanatory power at moderate levels.

**Table 5.** R Square and F Square.

Variables	R Square	F Square	
		Creative Skills	Lifelong Learning
Information Literacy		0.317	0.185
Creative Skills	0.487		
Lifelong Learning	0.356	0.242	

The explanatory impact of exogenous variables on endogenous variables can be detected through *the f*^2^ approach. The threshold value range for *f*^2^ is between 0.02 < *f*^2^ _ 0.15, 0.15 < *f*^2^ _ 0.35, and *f*^2^>0.35 which means that the effect can be categorized as small, medium, and large effect respectively. The figures in Table 5 explain that the explanatory effect *f*^2^ value of information literacy to creative skills is 0. 317 indicating the medium effect. Similarly, the explanatory effect *f*^2^ value of information literacy to lifelong learning is 0.185 indicating a medium effect. Finally, the explanatory effect *f*^2^ value of lifelong learning to creative skills is 0242 indicating a medium effect.

### Descriptive analysis

The descriptive statistical analysis was used to measure the levels of information literacy, lifelong learning, and creativity of the medical students. The scale of information literacy was divided into seven levels (1–7) whereas the scales for lifelong learning and creative skills were divided into five levels (1–5) from lowest to highest. The medical students had good levels of information literacy, lifelong learning, and creativity with a range of mean scores from 3.771 to 5.396. These mean scores showed that these medical students had reasonable levels of perception about their information literacy, lifelong learning, and creativity.

### Structural model

This study deployed SmartPLS statistical software to measure the interrelationships between information literacy, lifelong learning, and creative skills. PLS is a variance-based SEM technique facilitating the concurrent assessment of the measurement model. This approach analyses the reliability, validity, and structural and hypothesized relationships among the constructs used in the research model. Table 7 indicates the direct and indirect influence of information literacy on creative skills.

The results indicated that information literacy has a positive and significant effect on creative skills (β = 0.439, *p* < 0.05), which supported the research hypothesis H1. Similarly, information literacy has a positive and significant influence on lifelong learning (β = 0.395, *p* < 0.05), which accepted the research hypothesis H2. Furthermore, lifelong learning also appeared to be the positive and significant predictor of creative skills (β = 0.386, *p* < 0.05), which also supported the research hypothesis H3 (See Table 7 and Figure 2).

### Mediating effect

The indirect relationship between information literacy and creative skills through lifelong learning was also measured in the present study. The figures in Table 7 also indicated the positive but significant relationship between information literacy and creative skills through the mediating role of lifelong learning (β = 0.152, *p* < 0.05), which also supported the research hypothesis H_1_’. In addition, the two control variables, namely, geographical background and CGPA (Cumulative Grading Point Average) direct effect on creative skills were also measured. The figures indicated that both the geographical background (β = −0.037, *p* > 0.05) and CGPA (β = 0.031, *p* > 0.05) did not have statistically significant effect on creative skills (See Figure 2).

## Discussion

This study investigated the influence of information literacy on creative skills through lifelong learning among medical students of Khyber Pakhtunkhwa, Pakistan. The proposed research model was tested empirically based on PLS-SEM analysis. The descriptive statistics revealed that the information literacy (*M* = 5.396, SD = 1.1863), lifelong learning (*M* = 3.872, SD = 0.7886), and creative skills (*M* = 3.771, SD = 0.7243) of medical students were not at the optimum level (Table 6). These results were anticipated as the information literacy instruction programs in the medical libraries of Pakistan were in the infancy stage as most of the medical libraries used library orientations, bibliographic instructions, and occasional lectures as a method of information literacy delivery [54,55]. These results were in line with that of Ganesan and Gunasekaran [56] and Panahi et al. [4] who also reported that the information literacy skills of medical students were at a minimal level than expected. These findings were also supported by Ullah and Ameen [57] reporting that the Pakistani medical librarians also perceived the information literacy skills of medical students as inadequate. However, these results contradicted partially that of Basit et al. [58] who reported that the medical students from Sheikh Zayed Medical Complex were well information literate for basic skills levels and less comfortable with advanced skills. Similarly, these results disagree with that of Bashorun et al. [59] who discovered that Nigerian medical students have developed a varying degree of identifying, accessing, and using electronic information resources.

**Table 7.** Direct and indirect relations.

Hypothesis	Relations	Coefficient	Mean	SD	t	Decision
H1	IL -> Creative Skills	0.439*	0.441	0.048	9.238	Supported
H2	IL -> Lifelong Learning	0.395*	0.401	0.046	8.601	Supported
H3	Lifelong Learning -> Creative Skills	0.386*	0.386	0.054	7.184	Supported
H1’	IL -> Lifelong Learning -> Creative Skills	0.152*	0.155	0.028	5.378	Supported
**Control Variables**	Background -> Creative Skills	−0.037	−0.037	0.034	1.085	Not Supported
	CGPA -> Creative Skills	0.031	0.031	0.029	1.042	Not Supported

*P<0.001.

**Table 6.** Descriptive statistics.

Variables	N	Minimum	Maximum	Mean	SD
Information Literacy	473	1	7	5.396	1.1863
Lifelong Learning	473	1	5	3.872	0.7886
Creative Skills	473	1	5	3.771	0.7243

A closer look at the results revealed that the information literacy of medical students had a direct and positive effect on their creative skills (H1). This finding was anticipated as the creativity process undertakes certain information related task such as finding, accessing, using, managing, and creating information. These findings were consistent with that of the previous studies reporting the positive relationship between information literacy and creativity [19,21–23]. Likewise, Chang and Hsu [60] also reported information literacy as a positive predictor of employees’ creativity. In addition, some other studies from the workplace context also had similar results [60–63]. This finding also corroborated the outcomes of a recent and similar study by [16] who reported that digital literacy predicts positively creative thinking disposition. Similarly, the study by Naveed et al. [64] also reported the positive impact of information literacy one creativity among Pakistani journalists. Hence, the higher information literacy capabilities increase creative skills in the context of academia.

The PLS-SEM analysis also revealed that the information literacy of these medical students had a positive and significant influence on their lifelong learning skills (H2). This finding appeared to agree with those of the previous studies from the academic context that information literacy had a positive effect on lifelong learning [25,32,65]. Likewise, the study of Feng and Jih-Lian [31] also reported similar results that the information literacy of university teachers in Fujian Province, China affected positively their lifelong learning. Similarly, Solmaz [33] found that information literacy has a positive and significant association with lifelong learning skills among physical education and sports teacher candidates. In a recent study by Kesici [16], a somehow similar outcome was reported that the digital literacy of Turkish teachers influences positively their lifelong learning disposition. Likewise, Naveed et al. [64] also reported information literacy as a predictor of lifelong learning among journalists in Pakistan. Thus, it may be concluded that information literacy skills improve lifelong learning in the academic context.

The direct effect of lifelong learning of medical students on their creative skills was also investigated in this study. There was a statistically significant but positive connection between lifelong learning with creative skills (H4). This result was also expected as the production of distinctive and original ideas requires a lifelong and continuous learning effort [34]. Some other scholars also argued that creative skills can be developed through both formal and informal learning [35,36]. This finding was also supported by the outcome of Kesici [16] who found that the creative thinking disposition is affected positively by lifelong learning disposition among Turkish teachers. Thence, the higher levels of lifelong learning of medical students increase their creative skills.

The mediating role of lifelong learning in the relationship between information literacy and creative skills was also investigated. The outcomes revealed that lifelong learning played a statistically significant mediated role in the relationship between information literacy and creative skills (H4). As far as the knowledge of the researchers, the mediating role of lifelong learning in the relationship between information and creative skills had been examined for the first time. The finding needed to be corroborated through future inquiries. However, this finding somehow corroborated the results of a related and recent study by Kesici [16] who reported the mediating role of life learning disposition in the relationship between digital literacy and creative thinking disposition. It can be concluded that information literacy lays the foundations for lifelong learning which ultimately enhances creative skills. It is plausibly suggested that the medical information specialists and academicians needed to make arrangements for information literacy education in medical colleges to enhance the creative skills of medical students.

### Theoretical and practical implications

This study also has certain theoretical and practical implications. Theoretically, there was a dearth of research on information literacy concerning creative skills and lifelong learning, particularly the mediated role of lifelong learning in the relationship between information literacy and creative skills among medical students as no such empirical study has appeared so far. Moreover, the tested research model paved the way for the researchers to test it with other samples and expand the model through mediating and moderating variables. Thence, it may be determined whether or not the relationships between these constructs are valid for students of other disciplines. Practically, the academicians need to incorporate information literacy related content in the curriculum of medical education so that an information literate, lifelong learners, and creative medical workforce may be prepared. The medical librarians need to consider organizing frequent information literacy sessions which would ultimately help them to perform well not only in academia but also in the workplace. The credit-bearing mandatory course may also be initiated through integrated efforts of medical educators and librarians for the information literacy education of medical students. These research results provide empirical evidence for designing a need-based information literacy instruction program for medical students in particular and the students from other disciplines in general. The representative of the Pakistan Medical Library Association should come forward to create awareness about the effectiveness of information literacy and the way it influences lifelong learning, creativity, and performance in academic settings.

### Research limitations

There were several limitations to this research. First, this study measured information literacy, creative skills, and lifelong learning through the self-assessment method. There is a possibility that medical students overestimated their skills more than their actual ones which could be the primary limitation of this research. Second, the medical students were recruited using a non-probability convenient sampling technique due to the non-identifiable population, time limitation, and accessibility issues. Consequently, there might be a sampling bias and we could not recruit a representative sample despite the large size of the sample. That is why, the results of this study should carefully be generalized. Third, this study collected data from students of only 22 medical colleges at Khyber Pakhtunkhwa. Therefore, this study did not claim to be the voice of all medical students. Last, the survey was conducted in the context of medical students from Khyber Pakhtunkhwa, Pakistan which might limit the generalizability of the conclusions to other geographical, cultural, and academic contexts. Therefore, the outcomes of this research are needed to be strengthened with more such empirical evidence in the future.

## Conclusions

The conceptualized research model (Figure 1) was empirically tested that affirmed the interrelationships of information literacy, creativity, and lifelong learning among medical students. The conclusions drawn based on the present research included as follows: First, there was a positive relationship between information literacy, creativity, and lifelong learning among medical students. Second, information literacy positively and directly predicted creativity and lifelong learning. Third, lifelong learning also positively predicted creative skills. Last, the information literacy of medical students also had an indirect but positive effect on their creative skills through the mediation of lifelong learning. In other words, the levels of creative skills of the medical students increased as their levels of information literacy and lifelong learning increased. These findings provided a pragmatic insight for academicians and medical librarians about the effective role of information literacy in academia and the way it might contribute to the development of medical professionals with creative and lifelong learning capabilities for medical entrepreneurship and innovation. These results had great implications for medical academicians, information specialists, practitioners, and researchers.

## Appendix

7 = Almost Always True, 6 = Usually True, 5 = Often True, 4 = Occasionally True, 3 = Sometime but Infrequently True, 2 = Usually Not True, 1 = Almost Never True.

**Table:**

S#	Statements	Scale						
**I Can:**								
1.	Define the information I need	7	6	5	4	3	2	1
2.	Identify a variety of potential sources of information	7	6	5	4	3	2	1
3.	Limit search strategies by subject, language and date	7	6	5	4	3	2	1
4.	Initiate search strategies by using keywords and Boolean logic	7	6	5	4	3	2	1
5.	Decide where and how to find information i need	7	6	5	4	3	2	1
6.	Use different kinds of print sources (i.e., books, periodicals, encyclopedias, chronologies, etc.)	7	6	5	4	3	2	1
7.	Use electronic information sources	7	6	5	4	3	2	1
8.	Locate information sources in the library	7	6	5	4	3	2	1
9.	Use library catalogue	7	6	5	4	3	2	1
10.	Locate resources in the library using the library catalogue	7	6	5	4	3	2	1
11.	Use internet search tools (such as search engines, directories, etc.)	7	6	5	4	3	2	1
12.	Use different kinds (types) of libraries	7	6	5	4	3	2	1
13.	Use many resources at the same time to make research	7	6	5	4	3	2	1
14.	Determine the authoritativeness, currentness and reliability [validity] of the information sources	7	6	5	4	3	2	1
15.	Select information most appropriate to the information need	7	6	5	4	3	2	1
16.	Identify points of agreement and disagreement among [information] sources	7	6	5	4	3	2	1
17.	Evaluate information critically	7	6	5	4	3	2	1
18.	Synthesize newly gathered information with previous information	7	6	5	4	3	2	1
19.	Interpret the visual information (i.e., graphs, tables, diagrams)	7	6	5	4	3	2	1
20.	Write research paper	7	6	5	4	3	2	1
21.	Determine the content and form the parts (introduction, conclusion) of a presentation (written, oral)	7	6	5	4	3	2	1
22.	Prepare a bibliography [references]	7	6	5	4	3	2	1
23.	Create bibliographic records and organize the bibliography	7	6	5	4	3	2	1

**Section C**: **Perceived level of life-long learning**

**Instructions**: Please respond to the following statements concerning your lifelong learning skills using the following key:

1= Strongly Disagree (SD); 2= Disagree (D); 3= Undecided (U); 4= Agree (A); 5=Strongly Agree (SA)

**Table:**

S#	Statement	SA	A	U	D	SD
^1 R^	I prefer problems for which there is only one solution.	5	4	3	2	1
2 ^R^	I feel I am a self-directed learner.	5	4	3	2	1
3 ^R^	I try to relate academic learning to practical issues.	5	4	3	2	1
4 ^R^	When I approach new material, I try to relate it to what I already know.	5	4	3	2	1
5 ^R^	It is my responsibility to make sense of what I learn at school.	5	4	3	2	1

**Section D**: **Creative Skills**

**Instructions**: Please respond to the following statements concerning your creative skills using the following key:

1= Strongly Disagree (SD); 2= Disagree (D); 3= Undecided (U); 4= Agree (A); 5=Strongly Agree (SA)

**Table:**

SN	Statements	SA	A	U	D	SD
1.	I am nearly the first to try a new strategy or idea among my classmates.	5	4	3	2	1
2.	I have the ability to solve problems which others find difficult	5	4	3	2	1
3.	I explore and secure finances required to implement new ideas	5	4	3	2	1
4.	I usually find new uses for existing methods or existing equipment	5	4	3	2	1
5.	I develop satisfactory plans and schedules for the implementation of new ideas	5	4	3	2	1
6.	I usually search out new technologies, processes, techniques and ideas	5	4	3	2	1
7.	I use existing information to develop ideas that are useful for my academic performance.	5	4	3	2	1
8.	I develop ideas, methods or processes that are both original and useful for my career.	5	4	3	2	1

## ਀

Thank you for precious time and participation.
